# Supplementary material for: The Protective Role of Glutathione against Doxorubicin-Induced Cardiotoxicity in Human Cardiac Progenitor Cells
Source: Int J Mol Sci. 2023 Jul 28;24(15):12070. doi: 10.3390/ijms241512070 (PMC10419046; doi:10.3390/ijms241512070)
Supplement: Supplementary file 1 [file ijms-24-12070-s001.zip › ijms-2476000-supplementary.pptx]

## Slide 1
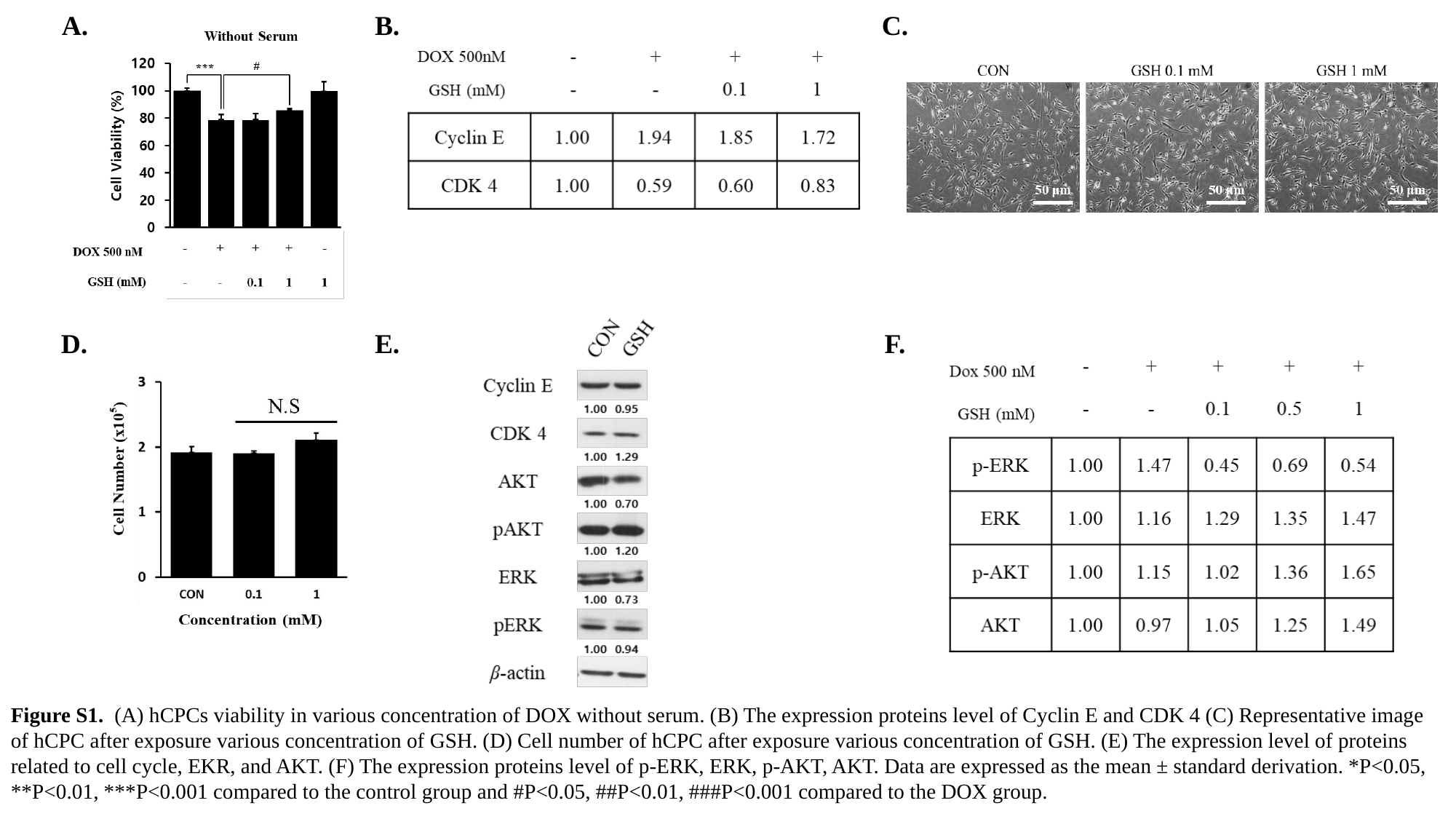

A.
B.
C.
D.
E.
F.
Figure S1. (A) hCPCs viability in various concentration of DOX without serum. (B) The expression proteins level of Cyclin E and CDK 4 (C) Representative image of hCPC after exposure various concentration of GSH. (D) Cell number of hCPC after exposure various concentration of GSH. (E) The expression level of proteins related to cell cycle, EKR, and AKT. (F) The expression proteins level of p-ERK, ERK, p-AKT, AKT. Data are expressed as the mean ± standard derivation. *P<0.05, **P<0.01, ***P<0.001 compared to the control group and #P<0.05, ##P<0.01, ###P<0.001 compared to the DOX group.

## Slide 2
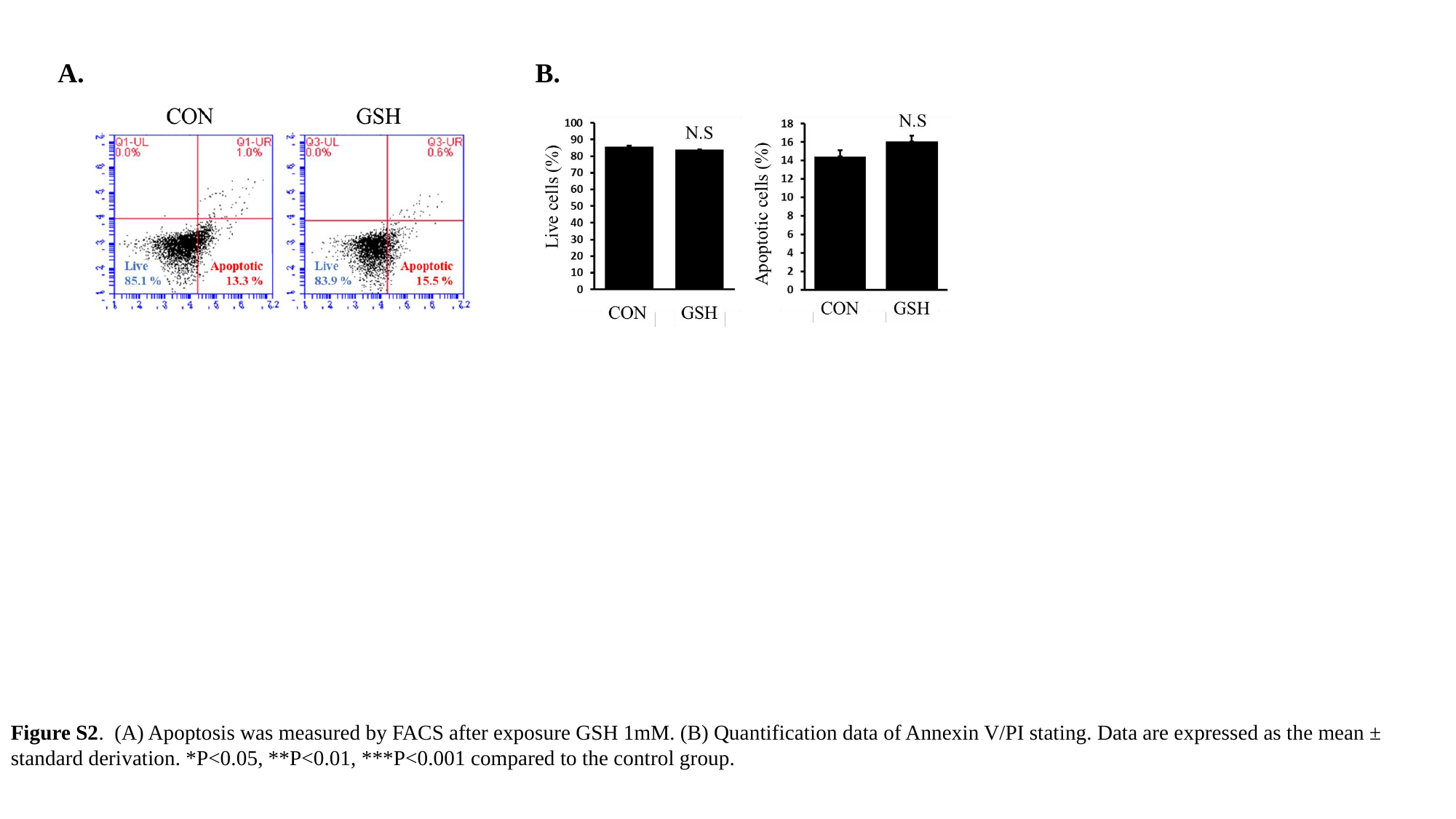

A.
B.
Figure S2. (A) Apoptosis was measured by FACS after exposure GSH 1mM. (B) Quantification data of Annexin V/PI stating. Data are expressed as the mean ± standard derivation. *P<0.05, **P<0.01, ***P<0.001 compared to the control group.
